# Crimean-Congo Hemorrhagic Fever Virus in Asia, Africa and Europe

**DOI:** 10.3390/microorganisms9091907

**Published:** 2021-09-09

**Authors:** Nariman Shahhosseini, Gary Wong, George Babuadze, Jeremy V. Camp, Onder Ergonul, Gary P. Kobinger, Sadegh Chinikar, Norbert Nowotny

**Affiliations:** 1 Centre for Vector-Borne Diseases, Canadian Food Inspection Agency, Lethbridge, AB T1H 6P7, Canada; nariman.shahhosseini@inspection.gc.ca; 2 Département de Microbiologie-Infectiologie et d’Immunologie, Université Laval, Québec City, QC G1V 0A6, Canada; gary.wong@crchudequebec.ulaval.ca (G.W.); gpkobinger@gmail.com (G.P.K.); 3 Institut Pasteur of Shanghai, Shanghai 200031, China; 4 Department of Biological Sciences, Sunnybrook Research Institute, University of Toronto, Toronto, ON M4N 3M5, Canada; george.babuadze@utoronto.ca; 5 Center for Virology, Medical University of Vienna, 1090 Vienna, Austria; jeremy.camp@meduniwien.ac.at; 6 Koç University, School of Medicine and Koç University Iş Bank Center for Infectious Diseases, Istanbul 34450, Turkey; oergonul@ku.edu.tr; 7 Department of Medical Microbiology, University of Manitoba, Winnipeg, MB R3T 2N2, Canada; 8 Department of Immunology, University of Manitoba, Winnipeg, MB R3T 2N2, Canada; 9 Department of Pathology and Laboratory Medicine, School of Medicine, University of Pennsylvania, Philadelphia, PA 19104, USA; 10 Pasteur Institute of Tehran, Tehran 1316943551, Iran; 11 Institute of Virology, University of Veterinary Medicine Vienna, 1210 Vienna, Austria; 12 Department of Basic Medical Sciences, College of Medicine, Mohammed Bin Rashid University of Medicine and Health Sciences, Dubai 505055, United Arab Emirates

**Keywords:** CCHF, epidemiology, tick, host, outbreak

## Abstract

The global spread of ticks and various tick-borne viruses (TBVs) suggests the possibility of new tick-borne diseases emerging. Crimean-Congo hemorrhagic fever virus (CCHFV) is an emerging TBV of the *Nairoviridae* family that causes serious disease that can be fatal in humans. CCHFV endemic foci can be found in Africa, Asia, the Middle East, and South-Eastern Europe, and has spread to previously unaffected regions and nations, such as Spain, over the last two decades. In this review, we discuss the current situation of CCHFV in Asia, Africa and Europe based on existing knowledge, and we discuss driving factors in the distribution and transmission of the virus, such as the spread of tick vector species and host reservoirs.

## 1. Introduction

Crimean-Congo hemorrhagic fever virus (CCHFV) is a tick-borne virus that causes moderate to severe hemorrhagic disease (Crimean-Congo hemorrhagic fever, CCHF) in humans with high case fatality ratios (up to 40%). The name is derived from the regions, where it was first reported (Crimea, 1945, and Congo, 1956). In terms of antigenicity, virus isolates from the two regions were indistinguishable [1]. CCHFV is transmitted to humans by bites of infected ticks (mainly species of genus *Hyalomma*) [2], but may also be transmitted to humans through contact with blood or tissue of infected animals, including human-to-human transmission in nosocomial settings [3]. The various vector species and the virus are widespread throughout the world, and endemic foci are absent only from North America, South America, and Australia. The transmission activity and distribution are dynamic, and both the virus and vector species have been introduced into new regions in recent years. In this review we provide a country-by-country summary of the known distribution of the virus based on vector surveys, host surveys, and human incidence reports. We discuss the impact on public health that CCHF has in endemic countries, and the potential impact that the spread of CCHFV may have in expanded or introduced regions.

## 2. Etiological Agent and Biology

CCHFV is a member of the *Orthonairovirus* genus, family *Nairoviridae*, and order *Bunyavirales*. The 41 species of the genus are classified into seven serogroups based on antigenic relationships [4]. The CCHFV serogroup contains CCHFV, Hazara virus from Pakistan, and Khasan virus from eastern Russia. In addition to CCHFV, Nairobi sheep disease virus (NSDV) and Dugbe virus are the only members of the genus known to be pathogenic for humans. NSDV, a tick-borne virus (TBV) of sheep and goats that causes intermittent benign illness in humans in East Africa, is thought to be similar to Ganjam virus in India [5]. Dugbe virus is a TBV that is generally associated with mild infection in cattle and sheep in West Africa and causes benign human disease on rare occasion [6]. The nairoviruses were originally classified based on their antigenic relatedness; however, the groupings have later been substantiated through the demonstration of morphological and molecular affinities between the viruses [7] and through comprehensive full-length sequence analysis [8]. Namely, CCHFV, NSDV, Hazara virus, Dugbe virus, and Kupe virus likely form a genogroup, yet formerly were divided into two serogroups (CCHFV serogroup and NSDV serogroup) [8]. CCHFV appears spherical under an electron microscope, with a dense core (capsid) surrounded by a lipid envelope and protruding spikes, and a diameter of around 100 nm [4].

## 3. CCHFV Genome

The viral genome is trisegmented and is comprised of single-stranded negative sense RNA. The three genomic segments of CCHFV have conserved 3′ end sequences of 3′AGAG (A/U) UUCU and a complementary 5′ end sequence, allowing the vRNA to form a looped structure. The small (S) segment (approximately 1.6 kb) has a single open reading frame encoding the nucleocapsid (N) protein. The medium (M) segment (approximately 5.4 kb) encodes a large glycoprotein precursor (GPC), which is processed into the two transmembrane surface glycoproteins (Gn and Gc) and several non-structural proteins. The large (L) segment (~12.1 kb) encodes a single L protein of approximately 460 kDa—the viral RNA-dependent RNA polymerase. Virus invasion of cells is most likely accomplished by clathrin-mediated endocytosis mechanism, followed by fusion of the viral envelope with endosomal membranes. Since monoclonal antibodies targeting Gc can neutralize CCHFV infection in mammalian cells, Bertolotti-Ciarlet et al. suggested that Gc is responsible for binding to cellular receptors [9]. All stages of viral replication happen in the cell cytoplasm, although little is known of this mechanism. Similar to many enveloped viruses, CCHFV surface glycoproteins are highly N-glycosylated, and glycosylation is important for several protein functions, such as folding, cellular transport and virus infectivity [10].

As CCHFV is a negative-sense RNA virus, the first step in replication is the transcription of the viral genomic RNA (vRNA) into positive-sense complementary RNA (cRNA). Viral messenger RNA (mRNA) is formed from cRNA by cap-snatching from host mRNAs, and the positive-sense cRNA also functions as a template for genomic vRNA. Virion assembly occurs in the Golgi complex [11]. Genetic reassortment might occur in CCHFV, especially in endemic areas where several CCHFV variants are circulating at the same time [12]. Further, there is evidence of genetic recombination in the CCHFV genome, which increases the diversity of the CCHFV genome [13,14].

The three ribonucleoprotein segments, with RdRP attached, acquire their outer viral glycoprotein-rich envelope by budding into the Golgi lumen. The virions are then transferred to the cell membrane and released from the infected cell by exocytosis [15]. The environmental stability of CCHFV is unknown, but the enveloped virions are susceptible to lipid solvents, and infectivity is destroyed by low concentrations of formalin and beta-propiolactone. Although the virus is labile in infected human tissues after death, analysis of human patient specimens tends to show that infectivity is maintained for at least a few days in separated serum at room temperature. Autoclaving destroys infectivity, however the virus remains stable at temperatures below 60 °C [16].

CCHFV replicates in various cell lines, such as Vero, CER, SW13 and BHK21, but does not usually yield high titers [17]. The virus is poorly cytopathic in cell culture, so titers may be demonstrated by indirect immunofluorescence in infected cells [18]. Historically, CCHFV has been isolated and titers have been determined most frequently by intracerebral inoculation of suckling mice [19].

## 4. Worldwide Burden and Phylogenetics of CCHFV

CCHFV is widely spread around the world: Asia (Iran, Afghanistan, Pakistan, Iraq, United Arab Emirates, Kuwait, Oman, Saudi Arabia, China, Tajikistan, Uzbekistan, Kazakhstan, India) [20], Africa (South Africa, Egypt, Mauritania, Kenya, Sudan, Democratic Republic of Congo, Chad, Niger, Nigeria, Senegal, Uganda, Tanzania) [21], and Europe (Albania, Bulgaria, Turkey, Greece, Georgia, Russia, Kosovo, Spain) [22]. Until now, CCHF has never been reported in northern Europe, Australia or in the Americas.

CCHF has the highest sequence diversity of any arbovirus, with divergence of 20%, 31%, and 22% for the S, M and L segments, respectively [23]. However, the divergences have little impact on the amino acid sequences, and the majority of M segment diversity is found inside the mucin-like domain. Next generation sequencing (NGS) has revealed that CCHFV quasispecies diversity is higher in the tick than in the mammalian host, and the distribution of single nucleotide variants across the genome varies between the mammalian host and the tick vector [24]. However, it should be noted that a heavily mouse-passaged virus could show less variations in immunocompromised mice but will show remarkable variations when reintroduced to the tick. Furthermore, a recent study revealed a single tick-specific amino acid substitution in the viral glycoprotein region that significantly lowered the viral fusion activity in human cells, indicating that a glycoprotein precursor variant found in ticks has greatly diminished function in human cells [25].

**Figure 1:**
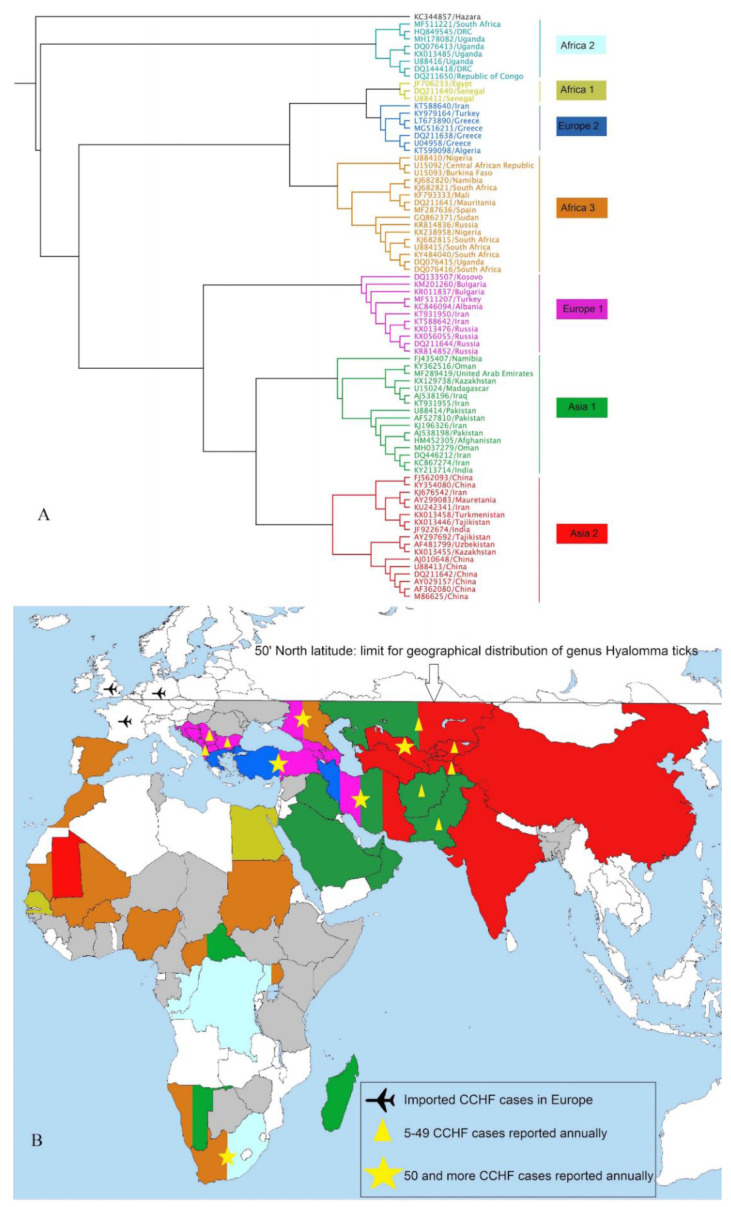
A Kimura 2-parameter model was selected to construct the phylogenetic Maximum Likelihood tree based on the S-segment of CCHFV using Geneious software V 11.0.5. The number of bootstraps was set at 1000 replications. The phylogenetic tree shows clustering of CCHFV strains in seven clades (**A**). Countries with one or more CCHFV strains are colored based on phylogenetic tree (Figure 1A). Countries with serological evidence for CCHFV are colored in grey. Imported CCHF cases to non-endemic countries in Europe are demonstrated (**B**).

The extensive sequence diversity of CCHFV in an area may increase the likelihood of the emergence of recombinant viral strains, as well as adaptation and circulation into new geographic regions [13]. Phylogenetic analysis of the CCHFV S-segment suggests the strains cluster into seven major clades: clade I (Africa 2), clade II (Africa 1), clade III (Europe 2), clade IV (Africa 3), clade V (Europe 1), clade VI (Asia 1) and clade VII (Asia 2) (Figure 1A) [26].

### 4.1. Trends in Asia

#### 4.1.1. Iran

Chumakov was the first to document CCHF in Iran in 1970, when 45% of sheep sera tested positive for CCHFV [19]. Then, in 1978, CCHFV was isolated from engorged *Ornithodorus lahorensis* soft ticks (Argasidae) in Northeastern Iran [27]. Since 1999, Iran has been actively detecting CCHF cases every year (mainly in southeastern Iran), with some CCHF cases being nosocomial. For example, some nosocomial infection cases were detected in the northeast of Iran in 2009, 2011 and 2012 [3,28].

Fever, hemorrhagic presentations, nausea, and myalgia were the most common clinical manifestations among Iranian patients. Petechiae, gastrointestinal (GI) bleeding, gingival bleeding, and epistaxis are common in those with hemorrhagic presentations. Further, thrombocytopenia was seen in the majority of CCHF cases in Iran. Furthermore, while there is no substantial link between the history of tick bites and fatal outcomes, there is a link between the history of livestock contact and death. According to statistical analysis, coma was reported in 3.2% of fatal cases. In Iran, diarrhea, hemorrhage, coma, unconsciousness, myalgia, icterus, and proteinuria were all found to be predictors of serious disease [29].

High risk professions for CCHF are slaughterers (abattoir workers), butchers, farmers, and housewives, while the death ratio is highest amongst housewives followed by farmers, and slaughterhouse workers and butchers [30]. In Iran, CCHF infection was mainly reported in men, approximately three times more than in women. An explanation would be gender differences in career choice, since almost all slaughterers and butchers in Iran are men. Although patients are mainly men, death ratios are high in women. Regarding age distribution, most Iranian patients are between 20–40 years. The number of CCHF cases usually rises during June and July [31]. Concerning molecular epidemiology of CCHFV, there are currently four different CCHFV genotypes circulating in Iran, including Asia-1, Asia-2, Europe-1 and Europe-2 [32,33,34,35,36,37] (Figure 1B).

#### 4.1.2. Afghanistan

*Hyalomma* ticks are common in Afghanistan. In the first documented record of CCHF in Afghanistan, 19 confirmed cases (two died) were reported in Takhar province (Northeast Afghanistan) in 1998. In 2008, the first multifocal outbreak was reported from Afghanistan [38]. In 2009, a seroprevalence analysis in the Engil district (Herat city) revealed that 11.2% of humans and 75% of livestock had previously been exposed to CCHFV [39,40]. Furthermore, from March 2017 to December 2018, a serosurvey study found that the average age of CCHFV positive cases was 30 years. The case fatality rate in this study was 21.6%. In terms of seasonal prevalence, the highest number of cases was found from June to September, which coincides with Eid-al-Adha. Butchers (13.7%) and shepherds (11.8%) are two high-risk occupations for CCHF in Afghanistan. In recovered cases, the average period from beginning of symptoms and admission to the hospital was 4.9 days, whereas in fatal cases it was 4.7 days [41].

#### 4.1.3. Pakistan

The virus was first isolated from *Hyalomma* ticks in Pakistan in the 1960s [42], while the first human case was reported in Rawalpindi in 1976, when a laparotomy was performed on a patient with abdominal pain, haematemesis and melaena [43]. In 1978, the first CCHF case was reported in Baluchistan province, and since then several outbreaks have been reported in this endemic area [44,45]. Since the year 2000, at least 14 sporadic outbreaks have been reported from Pakistan, while nine outbreaks occurred in Baluchistan province [46]. Several other provinces in Pakistan also deal with annual CCHF cases. For example, CCHF cases with 44% and 33% fatality were reported in the Punjab province in 2002 and 2003, respectively [47]. The most common CCHFV strain responsible for outbreak events in Pakistan was clade Asia-1 [48] (Figure 1).

#### 4.1.4. Iraq

CCHF is endemic in Iraq. CCHF in Iraq was first reported in 1979 [49]. In 1980, a seroprevalence study determined previous exposure to CCHFV among animals in three different faunal areas in Iraq, where 57.6% of sheep, 49.64% of goats, 29.28% of cattle, 58.73% of horses, and 23.23% of camels were found to be serologically positive [50]. The annual number of confirmed CCHF cases varied from zero to six between 1998 and 2009. In 2010, 11 confirmed and 28 suspected cases were reported. A case fatality ratio of 36% among confirmed cases has been reported in Iraq [51]. Sporadic cases and outbreaks of CCHF have occurred in Iraq including several nosocomial reports, e.g., (i) in 1979, two confirmed fatal cases (one physician, one nurse), (ii) in 1992, two confirmed cases (physicians), and (iii) in 1996, one confirmed case (physician) [52].

#### 4.1.5. United Arab Emirates

CCHF was first reported from the United Arab Emirates (UAE) in Dubai, 1979, when an index case and five secondary nosocomial cases occurred. Cattle imported from Iraq, Kenya, and Pakistan were thought to be the source of infection at the time. Until November 1993, no autochthonous cases of CCHFV infection had been identified in the UAE [53]. Between 1994 and 1995, 35 primary cases of CCHF were reported, with a case fatality ratio of 62%. The majority of the cases involved workers of livestock markets, butchers, and animal skin processors [54,55]. Recently, a novel lineage of CCHFV was detected in dromedary camels (*Camelus dromedarius*) and camel ticks (*Hyalomma dromedarii*) with potential reassortment of the M segment of the genome [56,57,58].

#### 4.1.6. Kuwait

A serological investigation in two hospitals in Kuwait showed that 4% of human sera collected between 1979 and 1982 were positive for anti-CCHFV antibody [59]. Since then, there are no other published data available on CCHFV circulation in Kuwait.

#### 4.1.7. Oman

CCHF was diagnosed in four patients by clinical presentation in 1995. Later, samples of imported and domestic animals were tested serologically for exposure to CCHFV, and 22% of samples reacted CCHFV IgG positive. Screening of 235 tick pools for CCHFV antigen showed that 19 tick pools were positive, sixteen of which were identified as *Hyalomma anatolicum* [60]. A study in 2016 confirmed serological evidence (IgG) of animal exposure with CCHFV [61]. A later seroprevalence study categorized butchers as a job with high potential for exposure to CCHFV [62].

#### 4.1.8. Saudi Arabia

CCHF was not diagnosed in Saudi Arabia until 1990, when an outbreak of viral hemorrhagic fever affected seven abattoir workers in Mecca, in western Saudi Arabia [63]. In Mecca, serosurveys of abattoir workers detected 40 confirmed or suspected cases (12 deaths) of CCHF between 1989 and 1990. It has been speculated that CCHFV had been introduced to Saudi Arabia by infected ticks on imported sheep arriving through Jeddah seaport [64].

#### 4.1.9. China

The first description of CCHFV in China was from the northwestern Xinjiang region in 1965 and the first isolation from patients and ticks (*Hyalomma asiaticum*) was in 1966 [65,66]. As CCHF cases have only been reported from Xinjiang, cases are known as “Xinjiang hemorrhagic fever” in China. This region provides an ideal habitat for the vectors, and human cases are reported during the active periods of the adult ticks, from March to June, primarily among males working outdoors. Antibodies to CCHFV have been detected in the serum of livestock and humans in other areas, implying transmission may occur elsewhere in mainland China. From 1965 to 1994, 260 CCHF cases were reported in China with a 21% case fatality ratio [65] with occasional epidemics (e.g., in 1997 with 26 cases and five deaths, and in 2001 with 51 cases and three deaths) [67,68,69].

#### 4.1.10. Tajikistan

CCHF is endemic in Tajikistan, and the first etiologically confirmed cases of CCHF were diagnosed in 1968. In Tajikistan, the average number of CCHF human cases recorded per year ranges from one to six, while epidemic years with over 20 cases have been reported on occasion (e.g., 21 cases in 1967; 26 cases in 2001; 29 cases in 2007; 37 cases in 2008; and 29 cases in 2009). According to some studies, cases associated with tick bites have a lower case fatality ratio (22%) than those associated with direct contact with contaminated blood (50%) due to differences in viral load. Men are twice as likely as women to develop CCHF, which is likely linked to occupational exposure to ticks or infected tissue/blood. Farmers, field workers, butchers, and medical personnel are among the most vulnerable groups. In Tajikistan, the average case fatality ratio was stated to be 24%. Sporadic outbreaks have been associated with nosocomial infections of hospital staff caring for an index patient who was infected via a tick bite, handling infected animal products, or unknown exposure routes [70].

#### 4.1.11. India

CCHF cases were reported for the first time in India from Gujarat in 2011. Four deaths were reported, three of which were from nosocomial transmission [71]. Additional nosocomial transmissions contributed to outbreaks of CCHF in 2012 and 2013 [72,73]. Phylogenetic analysis demonstrated that CCHFV strains from India fall into clade Asia 2 with the highest similarity to strains from Tajikistan (Figure 1A) [72].

#### 4.1.12. Turkey

In 2002, the first case of CCHF was identified in Tokat, Turkey. Cases with similar clinical and laboratory findings were reported from Tokat and its neighboring cities of Yozgat in the spring and summer of 2003 [74,75]. Since 2002, more than 10,000 confirmed cases were reported with an overall case fatality ratio of 5%. The majority of cases in Turkey were reported after 2000. Cases were widespread in many provinces of southern areas of the Black-sea region, central and eastern Anatolia, with rural areas accounting for 69.4% of cases. The male-to-female infection ratio was 1.13:1. The majority of CCHF patients in Turkey reported a history of a tick bites during the months of May, June, and July [76,77]. Two different circulating strains were detected in clades Europe-1 and Europe-2 [78] (Figure 1A).

#### 4.1.13. Georgia

Georgian public health officials have long suspected the presence of CCHF in the country, but laboratory confirmation using molecular diagnostic techniques was not possible until 2008. The first autochthonous case of CCHF in Georgia was discovered in 2009 in a suburb of the capital city, Tbilisi, and the virus was transmitted by tick bite [79]. The surveillance system reported 22 cases of CCHF from January to September 2014, the highest annual case count since 2009 [80]. During the years 2002–2007, CCHF patients in Georgia had a higher incidence of hemorrhagic symptoms than CCHF patients in neighboring Turkey (65% versus 23%, respectively). This could point towards a more pathogenic strain circulating in Georgia, or that milder cases were not identified [81]. Unfortunately, no detailed genetic or phylogenetic information on the viral strains in this area are available.

#### 4.1.14. Russia

Chumakov was the first to identify CCHFV in Russia, while investigating the agent of the first human outbreak in Crimea in 1944, when 200 military personnel were infected [82]. Since then, occasional epidemics occurred in Astrakhan between 1953 and 2005 (339 cases), in Rostov between 1963 and 2005 (377 cases), in Stavropol between 1953 and 2005 (263 cases), and in Krasnodar in 1948 (18 cases). Further, CCHF outbreaks and sporadic cases were recorded on several territories in the Southern Federal District in 2002 [83]. CCHFV isolates from Russia belong to the Europe-1 clade, but are distinct from those from Turkey and the Balkans. Further, a reassortment event has been identified in Russian CCHFV isolates [84].

### 4.2. Trends in Africa

#### 4.2.1. South Africa

The first case of CCHF was discovered in South Africa in 1981, when the virus was believed to have been introduced by infected tick vectors on migratory birds. However, antibodies to CCHFV were widely present in sera of livestock and wild vertebrates in South Africa, Zimbabwe, and Namibia, including sera that had been preserved for years [85]. Antibodies to CCHFV are very common in domestic and wild animals in South Africa, with lower prevalence along the southern coast and in the extreme northeast, where *Hy. truncatum* is the only *Hyalomma* species known in the region. Antibodies are most prevalent among large mammals including cattle, eland antelope, African buffaloes, giraffes, zebras, rhinoceroses, and ostriches, which are the preferred hosts of adult ticks, while CCHFV antibodies are most often found in hares among small mammals. Between 1981 and 2006, 44% of CCHF cases in southern Africa reported a tick bite, 40% reported contact with livestock blood or tissue, 12% of cases did not report any direct contact with livestock or tick bite but lived in or visited rural areas, and 4% of cases were infected nosocomially. During the same time frame, the majority of CCHF cases were men (83%) [86].

#### 4.2.2. Egypt

In a survey conducted between September 2004 and August 2005, 3.13% of 1022 animal sera tested positive for CCHFV-reactive IgG [87]. Although CCHFV is endemic in Egypt, the exact dissemination of the virus in human populations is uncertain [88]. A total of 4 infected cases and one death were recorded in Egypt in 1981 and 2012 [21].

#### 4.2.3. Senegal

In the 1970s, a preliminary study found CCHF infection in Senegalese livestock. CCHFV was later isolated from ticks obtained in a Senegalese abattoir. Since men are mainly involved in herding activities, where there is a high risk of tick bite (*Hyalomma truncatum*) or contact with infected animals, CCHFV infection is more common in men than in women in Senegal [89].

#### 4.2.4. Mauritania

CCHFV is endemic in southern Mauritania. In Mauritania, the first CCHF human case was identified and serologically confirmed in 1983. In May 1988, a fatal human case of CCHF was recorded in south-western Mauritania [90]. In 2003, six persons were admitted to the hospital with fever and hemorrhage; half of them died, and CCHFV infection was confirmed by serology. This was the first urban CCHF outbreak in Mauritania [91].

#### 4.2.5. Kenya

Evidence of CCHF in Kenya is limited. CCHFV was first discovered in *Rhipicephalus pulchellus* ticks obtained from a dying sheep in a veterinary laboratory in Kabete, Kenya, in the 1970s. Human CCHF was first recorded in Kenya in 2000, when a man with an acute hemorrhagic illness was admitted to a hospital in western Kenya [92].

#### 4.2.6. Sudan

Several suspected CCHF outbreaks, with evidence of both nosocomial and household transmission, and sporadic cases have been reported from the Kordufan region of Sudan. A serosurvey suggested that CCHFV is present in other areas of the country as well, but disease incidence is apparently lower [93]. In 2008, a CCHF outbreak was recorded in a Sudanese rural hospital, with a nosocomial chain of transmission [94]. CCHFV S segment sequences were most similar to those from South Africa, Mauritania, and Nigeria in clade 3, according to genetic analysis (Figure 1) [94,95].

#### 4.2.7. Madagascar

In 1985, the first CCHFV was discovered in *Rhipicephalus microplus* ticks collected on cattle in a slaughterhouse in Antananarivo as part of a tick screening study in Madagascar [96]. Further, phylogenetic studies showed that CCHFV strains from Madagascar were more similar to strains from the Middle East and Asia than to African strains. In 1988, evidence of CCHFV infection was detected in human sera collected in Mandoto. To determine the prevalence of CCHFV infection among high-risk groups (e.g., slaughterhouse workers) in different geographical areas in Madagascar, a national cross-sectional serologic survey was performed in 2008–2009. In contrast to similar findings in other endemic countries, the incidence of CCHFV infection among high-risk professionals was found to be low [97].

#### 4.2.8. Niger

A 1995 serosurvey study found that an archive of domestic animal sera collected in Niger between 1984 and 1988 were serologically positive for CCHFV, suggesting that the virus was circulating in the country [98]. However, no human cases were reported.

#### 4.2.9. Nigeria

A study found 24% of cattle and 2% of goats were serologically (IgG) positive for CCHFV in Nigeria in 2015 [99]. A large study in the human population in northeastern Nigeria provided evidence of active and prior exposure to CCHFV, and phylogenetic analysis of a human-derived CCHFV sequence obtained by NGS placed the strain in the Africa 3 clade [100].

#### 4.2.10. Ghana

Tick samples from *Hyalomma excavatum* and *Amblyomma variegatum* tested positive for CCHFV in a CCHF surveillance study in Ghana. Exposure to CCHF was evidenced by the presence of CCHFV-reactive IgG in human serum samples obtained from abattoir workers [101].

#### 4.2.11. Uganda

Since 2010, Uganda has established a surveillance system for viral hemorrhagic fevers. The surveillance system has resulted in the early detection of occasional CCHF outbreaks in humans, the majority of which have occurred in the central regions of Uganda. Anti-CCHFV antibodies are present and prevalent in cattle [102].

In Uganda, there were eight confirmed CCHF outbreaks between 2013 and 2017. In 2013, there was a CCHF outbreak in the Agago District, involving three patients. Less than ten cases were documented in the following years. In July 2018, the infection of two patients was confirmed by PCR in Isingiro District. In the next seven months, 13 CCHF cases were confirmed in different regions. However, it is unclear if the increase was attributable to improved surveillance or an actual increase in cases during this time period [103].

### 4.3. Trends in Europe

#### 4.3.1. Albania

An outbreak of eight cases of CCHF occurred in Albania during the spring and summer of 2001, with the infection of seven cases confirmed by laboratory tests. A nosocomial infection was discovered, as well as a familial cluster of cases. Genetic analysis revealed that the causative virus clustered together with other European CCHFV cases [104].

#### 4.3.2. Bulgaria

Between 1953 and 1974, numerous CCHF cases were detected in Bulgaria, and the death ratio was around 17%. During this time, 20 nosocomial infections were reported. Between 1975 and 1996, the number of reported CCHF cases decreased, with a death ratio also decreasing to 11.4%. The CCHFV strains from Bulgaria were found to cluster with other Balkan strains from Kosovo and Albania [105].

#### 4.3.3. Greece

In Northern Greece, the first fatal case of CCHF was discovered in 2008 [106]. A CCHFV strain (AP92) was isolated from *Rhipichepahlus bursa* ticks collected from goats in Northern Greece, in 1975. Antibodies to CCHFV were found in 6.25% of residents where strain AP92 was isolated; however, positive cases did not have any symptoms of CCHF. According to a survey conducted in Greece between 1981 and 1988, the seroprevalence of CCHFV was 1% among the population surveyed, and approximately 3% were positive for CCHFV-reactive IgG in a 2008–2009 study [107]. Since no CCHF cases had been identified in Greece, it was assumed that the human antibodies were against strain AP92, which appears to be moderately pathogenic to humans and thus a good candidate for vaccine research. The first serious case of CCHF in Greece was registered in 2008, when a woman died in Komotini, a town in northeastern Greece. Molecular analysis revealed that the causative strain (Rodopi) was genetically distinct from strain AP92 [108].

#### 4.3.4. Kosovo

The first cases of CCHF were reported in 1989. In 1995, 2001, and 2004, there were three major outbreaks with a total of 186 serologically confirmed cases [109]. The central and southwestern parts of Kosovo are hyper-endemic for CCHF [110]. A phylogenetic study suggested that CCHFV was recently introduced to Kosovo (within the last 50 years), sharing a common ancestor with strains from Turkey [111].

#### 4.3.5. Spain

In 2010, CCHFV was discovered in *Hyalomma* ticks in Cáceres, Spain [112]. In August 2016, two autochthonous CCHF cases were identified for the first time [113], although a retrospective study revealed one autochthonous case dating back to 2013 [114]. From 2016 to 2020, seven human cases with molecular confirmation were recorded in Spain, three of which were fatal [115]. The CCHFV strain in Spain is similar to a strain of CCHFV identified in Mauritania (Africa-3) [113]. Furthermore, recent observations of viral sequences identical to those found in eastern Europe (genotype V, Europe-1) in a patient and ticks from deer and wild boar raise the likelihood of CCHFV being introduced into Spain through the animal trade. The seropositive rates of animals found in southern Spain reflect an established tick-host-tick cycle in some regions, and segment reassortment found in a sequenced virus from one case suggests the virus has a great ability to adapt [115,116].

#### 4.3.6. Imported Cases to Europe

Travelers from CCHFV-endemic areas to non-endemic European countries have been recorded in the UK (returning travelers from Afghanistan, Bulgaria, Zimbabwe) [117,118,119], France (returning traveler from Senegal) [120,121] and Germany (returning travelers from Afghanistan and Bulgaria) [122,123].

## 5. Zoonotic Maintenance of CCHFV

### 5.1. Vectors

CCHFV vectors are mainly *Hyalomma* ticks, a genus of hard-bodied ticks in the family Ixodidae [124]. However, CCHFV was isolated from at least 35 different tick species, including soft tick species (family Argasidae). Most *Hyalomma* sp. are two-host ticks—immature stages (larvae and nymphs) feed on the same individual host before molting to the adult stage which then feeds on a second host—but three-host lifestyles have also been observed [125].

Immature stages and adults may feed on similar vertebrate hosts (e.g., *Hy. anatolicum* feeds on domestic mammals), whereas others may feed on distinct taxonomic groups (e.g., *Hy. marginatum* and *Hy. rufipes* immature stages feed on birds, hares and/or hedgehogs, and adults on cattle and other large mammals) [126]. Birds may play an important role in disseminating immature ticks, particularly those of the *Hy. marginatum* complex, and migrating birds may introduce infected ticks into new regions [127].

CCHFV may be passed from infected male to uninfected female ticks during mating and/or trans-ovarially from infected female ticks to their offspring. Transmission via co-feeding on the same host has also been demonstrated [128]. The tick remains infected trans-stadially, and may survive up to 2 years between bloodmeals, making ticks the zoonotic reservoirs of the virus, with the transiently-infected vertebrates serving as amplifying hosts. Various *Hyalomma, Rhipicephalus, Haemaphysalis,* *Dermacentor, Ixodes, Amblyomma,* and *Ornithodoros* species have been implicated in CCHFV transmission, depending on the biogeographic region, while *Hy. marginatum* is known as the main vector for CCHFV (Table 1) [124,129,130,131,132,133,134,135,136].

### 5.2. Vertebrate Hosts

CCHFV is present in a variety of wild and domestic animal species [137,157]. In particular, a high seroprevalence is often found in cattle, sheep, goats, and camels [158,159]. Horses, donkeys, pigs, rhinoceroses, giraffes, buffalos and other mammals (e.g., hedgehogs, hares, dogs, mice) have also tested positive for CCHV antibodies. The majority of tested bird species were shown to be CCHFV serologically negative; however, antibodies have been found in ostriches, and these animals have become viremic after experimental inoculation [160]. A blue-helmeted guinea fowl (experimentally infected), as well as magpies, red-beaked hornbills, and shiny starlings, were all found positive for the CCHFV genome and/or antibodies. Although immature *Hy. anatolicum* ticks can feed on reptiles, antibodies to CCHFV have only been reported from a tortoise from Tajikistan [161].

### 5.3. Transmission Modes and Environmental Amplification

Infection of humans and other mammals is systemic, and CCHFV can be found in blood, bodily fluids, and other tissues. In humans, horizontal transmission from mother to child has been confirmed [162,163]. The disease has not been linked to airborne transmission [46]. The two most common routes of human infection are direct transmission by contact with infected tissues or tick bites. In livestock, the infection is usually asymptomatic or may occasionally result in mild fever [164].

It is unclear whether maternal antibodies are protective against CCHFV infection in livestock [165]. In domestic ruminants, seroconversion ratios typically increase with age, and there is evidence that animals become infected at a very young age. Consequently, humans may become infected if they come into contact with viremic blood from young animals when conducting procedures including castrations, vaccinations, or ear tag insertion [19,166]. The evidence suggests that humans may acquire the infection by contact with viremic blood on broken skin, which is consistent with reports of nosocomial infection among health care staff [19].

**Figure 2:**
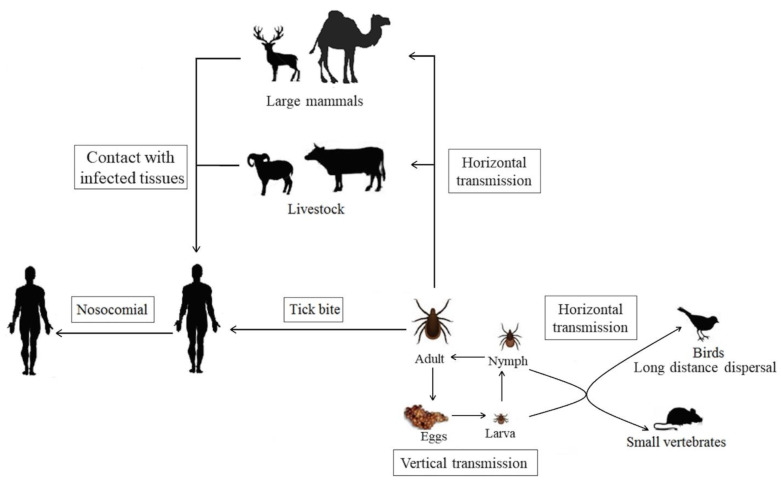
The transmission cycle of CCHFV. The boxes show transmission routes.

In light of the serological data, it is often surprising that livestock infection occurs on a large scale in areas infested by *Hyalomma* ticks, whereas evidence of human infection is much lower. Despite the fact that a large percentage of patients report tick bites, humans are not the preferred hosts of *Hyalomma* ticks and are bitten less often than livestock (Figure 2) [167]. Additionally the duration of viremia in livestock is short and of low intensity, which also limits the spillover potential from livestock to humans. It is also worth noting that a considerable proportion of CCHFV infections may be subclinical [168].

## 6. Factors Affecting the Range Expansion and Introduction of CCHFV

The introduction of CCHFV from an endemic area to a non-endemic area or the introduction of new viral strains into a previously endemic area is made possible by the relatively long attachment of ticks to their animal hosts, the level of infestation of an animal with tick species, and the movement of animals via livestock trade [169,170]. This is suspected to have happened with other tick vectoring viruses, such as the introduction of *Ha. longicornis* (a vector for severe fever with the thrombocytopenia syndrome virus) from China to the US by animal trade [171,172]. In Europe, the transportation of infected ticks on migrating birds presents the most likely route of introduction for CCHFV.

Using a retrospective modeling approach, in 2015 Estrada-Pena et al. provided convincing data that predicted the increased survival of questing stages throughout most of Europe, with a non-negligible increase in the developmental rate in many areas. Their analysis suggested that large patches of Europe (e.g., the Rhine valley in France, Belgium, Netherlands, and Germany; eastern and southeastern Austria, northern Hungary, and western Slovakia; most of Spain and France; and a large part of Ukraine) are becoming more suitable for the survival of *Hy. marginatum* [173]. A more recent model, incorporating migratory birds, shows that these same areas have the highest probability of introduction of *Hy. marginatum* [174]. In fact, 2018–2019 was a particularly intense year of recorded introductions of *Hyalomma* sp. into Europe, and was noted to be a very warm year (and an epidemic year of mosquito-borne West Nile virus in Europe): Twenty *Hy. marginatum* and *Hy. rufipes* identified on humans, horses, or cattle in Sweden in 2018 with no history of travel outside of Sweden in the previous two months [175]. Some were positive for *Rickettsia aeschlimannii.* The data suggest that the adult ticks developed from immature stages brought into the country on migratory birds, and warm temperatures in 2018 allowed them to survive and mature to adults. The importation of ticks into Germany in 2018 was very similar to the situation in Sweden, wherein 35 ticks were observed on humans or livestock (sheep, horses). Based on descriptions, many were likely *Hyalomma* species, with 10 confirmed to be *Hy. marginatum* and 8 *Hy. rufipes*. Half were positive for *R. aeschlimannii* and negative for other pathogens. Again, migratory birds were assumed to be the most likely route of introduction of immature specimens, and the favorably warm summer provided conditions necessary for their development to adult stages. An adult *H. marginatum* was reported in eastern Austria in 2018, collected from a horse; it was also positive for *R. aeschlimannii* [176]. There is evidence of the continued introduction of *Hy. marginatum* and *Hy. rufipes* into France via migratory birds, although *Hy. marginatum* is likely established along the Mediterranean coast of France [177,178]. In 2019, *Hy. rufipes* was collected from a horse in the southern Czech Republic, with anecdotal evidence of another *Hyalomma* sp. specimen observed at the same location in 2018 [179]. The publication by Hubálek et al. provides a list of reported introductions of *Hy. marginatum* or *Hy. rufipes* ticks in Central Europe—all but two specimens were reported in the last 10 years [179]. Due to global climate change, the 50° north latitude as a geographical limit for *Hyalomma* ticks may become increasingly inaccurate.

*Hy. marginatum* is established along the entire Mediterranean Sea coast of Europe (Figure 1B), although the reason for the absence of CCHFV from some of these regions (e.g., Italy, Croatia) is unclear. The detection of CCHFV in *Hy. lusitanicum* in Spain in 2010 preceded the first prospective and retrospective human cases from 2016 and 2013, respectively [112,114,180], however it is more likely that *Hy. marginatum* was the initial source of the introduction of CCHFV. As illustrated by the established transmission of CCHFV in Spain, the importation of *Hy. marginatum* on migrating birds presents an annual risk of the importation of tick-associated pathogens to north and central Europe, including CCHFV. The ECDC publishes annual or semi-annual reports on the distribution of *Hy. marginatum* (https://www.ecdc.europa.eu/en/publications-data/hyalomma-marginatum-current-known-distribution-march-2021, accessed on 7 September 2021). In contrast to Europe, North America and Australia have comparatively reduced risks of importation of CCHFV, *Hyalomma* ticks, or both. While sporadic introductions of *Hyalomma* spp. have been recorded, mostly on animals or animal products, the frequency of importation is much lower than in Europe [181,182].

Additionally, the animal trade may be a source of potential transportation either via infected animals or infected ticks on animals. While there have been no definitive reports of transportation of CCHFV to new regions via animal trade, the phylogenetic (Figure 1A) and phylogeographic analyses suggest that recent distribution of CCHFV genotypes is the result of livestock trade [183,184]. Evidence of reassortment and recombination are more suggestive of short-distance mixing of various genotypes. However, a recent serosurvey in the United Arab Emirates showed that camels at a livestock market had the highest ratio of seropositive animals, compared to camels at a family farm and at a recreational area [57,58]. This is congruent with the increased risk of exposure for animal care workers and abattoir workers, but also may increase the risk of long-distance transportation of CCHFV via infected livestock or ticks. This is an aspect of global CCHFV activity that should be studied further.

## 7. Prevention and Control

Tick bites and nosocomial infections are the main routes of transmission, while infection via contact with contaminated livestock tissues is a known risk factor for certain professions. For humans, tick bites can be mitigated by wearing clothing impregnated with pyrethroid acaricides [185]. Tick control for livestock, on the other hand, is impractical in many parts of the world where *Hyalomma* ticks are the most prevalent. In their review of control measures of ticks, focusing on *Hyalomma* sp., Kumar et al. (2020) conclude that the use of acaricides is likely unsustainable and largely inefficient. They review the development and efficacy of tick vaccines, and compare them to more sustainable tick management alternatives. Until alternatives are developed and become available, focused acaricide treatment of livestock may be effective in reducing tick populations in response to confirmed human infections. Limiting adult ticks on livestock would have the effect of reducing contact with virus amplifying hosts, and thus reduce the risk of human infection.

Occupational exposure may be limited by wearing gloves and avoiding exposure of skin and mucosal membranes to fresh blood and other animal tissues. These measures should be strictly followed by veterinarians, slaughter workers, and those working with potentially infected livestock [185]. As CCHFV causes severe disease, can be transmitted via exposure to tissues of viremic animals and/or humans, and no vaccines or specific therapeutics exist, CCHF is placed in a high biohazard class. This dictates that virus culture or experimental animal infections are permitted only in biosafety level 4 (BSL-4) laboratories and BSL-3 plus laboratories in endemic countries [186,187]. Nosocomial infections are most likely the result of needle stick injuries or broken skin contact with infected blood, tissues, and bodily fluids of infected patients. While aerosol transmission is not considered a primary mode of transmission, patients with CCHFV infection should be isolated and subjected to barrier nursing techniques before the diagnosis is confirmed in order to protect health care workers from infection [188]. Protective gear, such as disposable gowns, gloves, masks, goggles, and overshoes, should be worn by health care staff and discarded when they exit the isolation room through the anteroom. All products removed from the isolation ward should be properly disinfected or disposed of. Disinfectants such as 1% hypochlorite and 2% glutaraldehyde will inactivate CCHFV. For secure transportation, blood samples should be wrapped in absorbent material like paper towels and stored in secondary leak-proof containers like rigid metal or plastic screw-cap containers or sealed plastic bags [189].

In summary, the use of joint tick control measures with endemic countries, as well as awareness and training programs for health care workers and slaughterers (such as wearing protective clothing and gowns) and preventing illicit livestock transportation all play a critical role in disease control, especially in hot spot regions [190,191].

## 8. Outbreak Control and Response

Given the complexity of tick control, strategies should concentrate on improving surveillance and expanding laboratory capability in already endemic areas and areas at risk of CCHF expansion [192]. The early disclosure of the existence and extent of disease activity is of crucial importance so that effective containment measures can be taken. The late recognition and reporting of disease outbreaks results in continued transmission and delays the start of the clinical, epidemiological, and laboratory investigations that are required to provide the evidence needed to implement effective prevention and control methods. Surveillance teams should be established, consisting of locally recruited personnel who are trained on the spot and supervised by one or more epidemiologists. Both team members should be aware of the precautions. Their job is always to collect specimens to send to laboratories in order to confirm a suspected diagnosis and to administer emergency isolation measures. Therefore, they should be provided with standard forms for case management assessment. For the investigation and control of outbreaks, transportation facilities and a means of rapid communication are needed.

The first reports of an outbreak can come from epidemiological surveillance and early-warning systems, as well as other sources such as veterinary services, laboratories, or, as is often the case, public rumors spread quickly through the media. Corroborative investigations from various sources will reveal whether the evidence has any credibility. Further, a rapid site visit by clinicians and epidemiologists with relevant experience with the suspected disease might be necessary. It is critical that deployed experts are aware of all possible diseases associated with the rumor, and that laboratory specimens are obtained to confirm the preliminary clinical diagnosis. The initial data analysis may tend to show positive signs of a disease. However, differential diagnosis should be given high priority, with a sufficient number of cases investigated. Field operations should be launched as soon as the outbreak is confirmed. A consistent description of cases and contacts is needed for effective investigation and containment measures. The case definition’s terminology is important because it can aid the field investigation teams’ case-finding procedures. As a result, the case definition must meet two requirements: it must be precise but not too restrictive.

## 9. Vaccine Option

Despite current knowledge and expertise on CCHFV, successful prophylaxis and therapeutics are urgently needed to avoid major outbreaks. To this end, developing safe and effective human and animal vaccines is important to reduce the risk of zoonotic transmission. To date, CCHFV vaccine efforts have resulted in protection against lethal challenge in pre-clinical trials, but further research is required to improve these potential vaccines, as well as new alternative treatment options. Attenuated vaccines and non-viral recombinant DNA vaccines have shown promising results, but more basic virological research is required to improve our understanding of CCHFV biology and improve vaccine design.

Although a vaccine has been licensed in Bulgaria and is being used on a small scale in Eastern Europe, international regulatory approval is uncertain due to concerns about efficacy and side effects [193]. Recombinant candidate vaccines have been developed using the Modified Vaccinia Virus Ankara (MVA). For example, Buttigieg et al. showed that animals administered the MVA-glycoprotein vaccination had no clinical symptoms up to 14 days after the challenge [194]. In another study, Zhang et al. used the MVA platform to develop a vaccine that expressed the CCHFV nucleoprotein (NP, S segment), which induced an immune response but failed to protect animals against fatal disease in a challenge experiment [195].

## 10. Future Forecast

CCHFV has been regarded as a significant emerging public health threat, and it is becoming a higher priority for public health. According to the available data, CCHF outbreaks will continue to occur in endemic areas and have shown geographical expansion, especially in the southwest areas in Europe. CCHFV invasion to previously non-endemic areas can occur and has been documented, although established transmission in new regions may require virus adaptation to new vectors [155]. Climate change may alter diversity and composition of tick species in ecological niches, which can cause virus expansion to previously naïve areas for CCHF in the near future [112,180]. Given the presence of both the vector and the virus in southern Europe, we speculate the risk of CCHFV spreading to other European countries. Furthermore, there are currently no reports on CCHFV in Southeast Asia (i.e., Laos, Thailand, Cambodia, Vietnam, Myanmar, etc.). However, *Hyalomma* ticks are endemic in these countries and if the CCHFV is circulating in Yunnan Province in China (bordering these countries), it can be speculated that the virus is in Southeast Asian countries, but not investigated yet. Currently, Oceania and the Americas are the only zones free of CCHFV. However, it should be noted that North America is located on two avian migration routes connecting to CCHFV-endemic regions: the Pacific Americas flyway connecting Western North America with Eastern Asia; and the East Atlantic flyway connecting Eastern North America with Western Europe and Africa. Thus, CCHFV strains found in Eastern Asia or Western Europe and Africa have the potential to spread to North America, where many of the corresponding vector species are endemic [130]. It is possible that CCHFV may be introduced into North America in the future through intercontinental transmission of tick species, as it is suspected to have happened with CCHFV in Spain (introduced from Africa to Spain by migratory birds carrying infected ticks) [112,127,180].

The emergence of new virus strains as a result of mutation, recombination, and reassortment, particularly in endemic areas where multiple strains of CCHFV are circulating at the same time, will be a potential challenge for public health agencies [13,14,34]. Thus, rapid diagnostics play a critical role in the early detection and management of outbreaks. Further, early diagnosis of CCHF is critical for infection control and establishing early treatment of symptoms. Given CCHF strain variations, it is recommended that PCR be used in combination with immunological assays to achieve the highest sensitivity for diagnosis. In order to improve the use of existing assays for better screening and prompt diagnosis, a better understanding of CCHF viral and antibody kinetics across a wide range of sample types is needed. NGS can be an effective method to monitor circulating strains and potential viral mutations.

## Figures and Tables

**Table 1:** A list of tick species that have been confirmed CCHFV-positive in different regions.

Family	Genus	Species	Geographical Distribution	References
Ixodidae	*Hyalomma*	*Hy. marginatum*	Middle East, Northern Africa, Southern Europe	[130,131,132]
		*Hy. dromedarii*	Middle East, Northern Africa	[131,135,137]
		*Hy. rufipes*	Middle East, Africa	[92,137,138,139,140,141,142]
		*Hy. turanicum*	Asia, Africa	[140,143]
		*Hy. nitidum*	Central Africa	[139,144]
		*Hy. anatolicum*	Asia	[129,130,131,132,135]
		*Hy*. *asiaticum*	Asia	[129,131,132]
		*Hy. detritum*	Middle East, Africa	[131,143]
		*Hy. excavatum*	Africa, Middle East	[55,144,145,146]
		*Hy. truncatum*	Africa	[137,138,139,144]
		*Hy. schulzei*	Arabia peninsula	[131,147]
		*Hy. impeltatum*	Northern Africa, Arabian Peninsula	[55,138]
		*Hy. lusitanicum*	Africa, Spain	[133]
		*Hy. isaaci*	Africa	[140]
		*Hy. impressum*	Africa, Pakistan	[142,148,149]
	*Rhipicephalus*	*Rh. sanguineus*	Asia	[129,130,131,136]
		*Rh. bursa*	Southeastern Europe, Middle East	[131,132,146]
		*Rh. annulatus*	Middle East, Central parts of Africa	[145,150]
		*Rh. turanicus*	Southern Europe, Asia	[142,143,146]
		*Rh. rossicus*	Caucasia, southern Russia	[19]
		*Rh. evertsi*	Sub-Saharan Africa	[91,151]
		*Rh. decoloratus*	Uganda	[152]
		*Rh. appendiculatus*	Iran	[153]
		*Rh. microplus*	Africa, Pakistan	[132,142,148]
		*Rh. guilhoni*	Senegal	[138,151]
	*Haemaphysalis*	*Ha. punctata*	Some parts of Asia, South-East of Europe	[131]
		*Ha. inermis*	Iran	[124,131]
		*Ha. concinna*	Turkey	[143]
		*Ha. sulcata*	Iran	[26]
		*Ha. parva*	Turkey, North Caucasus	[145,154]
	*Dermacentor*	*De. marginatus*	Southern Europe, Middle East, Mediterranean	[19,131,146]
		*De. niveus*	Tajikistan	[132]
	*Ixodes*	*Ix. ricinus*	Europe, Mediterranean, Northern Africa	[155,156]
	*Amblyomma*	*Am. variegatum*	Sub-Saharan Africa	[138,151]
Argasidae	*Ornithodoros*	*Or. lahorensis*	Iran	[131]

## Data Availability

Data sharing is not applicable to this article.
